# Cionni-modified capsular tension ring for surgical repair of cyclodialysis after trabeculectomy: a case report

**DOI:** 10.1186/s12886-017-0582-4

**Published:** 2017-10-27

**Authors:** Qinghe Jing, Jiahui Chen, Junyi Chen, Yating Tang, Yi Lu, Yongxiang Jiang

**Affiliations:** 1 Department of Ophthalmology and Vision Science, Eye and ENT Hospital of Fudan University, 83 Fenyang Rd, Shanghai, 20031 China; 2 Key Laboratory of Myopia of State Health Ministry, and Key Laboratory of Visual Impairment and Restoration of Shanghai, Shanghai, China

**Keywords:** Surgical techniques, Cionni-modified capsular tension ring, Cyclodialysis, Ocular hypotension

## Abstract

**Background:**

To report a case for repair of cyclodialysis after trabeculectomy with Cionni-modified capsular tension ring.

**Case presentation:**

A 64-year-old man who had undergone trabeculectomy of his left eye 3 months earlier visited our clinic owing to blurred vision. His visual acuity was 20/2000 and the intraocular pressure (IOP) was 6 mmHg. Slit-lamp examination showed a shallow anterior chamber and dense cataract. Ultrasound biomicroscopy revealed 360 ° detachment of the ciliary body and suspected cyclodialysis of the trabeculectomy incision. Choroidal detachment was confirmed by B-scan ultrasonography and optical coherence tomography. Phacoemulsification was performed in which a foldable intraocular lens (IOL) was implanted in the capsular bag and a Cionni-modified capsular tension ring (MCTR) was inserted into the ciliary sulcus. The maximum focal point of the MCTR was rotated to the site of the most severe cyclodialysis and the MCTR was sutured to the sclera through its two eyelets. The patient’s best-corrected visual acuity improved to 30/50 and the IOP increased to 16 mmHg after surgery. Gonioscopy and ultrasound biomicroscopy confirmed closure of the cyclodialysis and resolution of choroidal detachment.

**Conclusions:**

Phacoemjulsification with implantation of an intraocular lens combined with insertion of an MCTR into the ciliary sulcus appears to be a relatively safe, effective, minimally invasive method for repairing cyclodialysis in cataract patients. Although the technique yielded good results and appeared to be safe in one patient, further studies are necessary to validate the findings on more patients and with a long-term follow-up.

**Electronic supplementary material:**

The online version of this article (10.1186/s12886-017-0582-4) contains supplementary material, which is available to authorized users.

## Backgroud

Cyclodialysis involves separation of the meridional ciliary muscle from the scleral spur [1], resulting in a communication channel between the anterior chamber and the suprachoroidal space [2]. Cyclodialysis may be due to trauma or have an iatrogenic cause, and occurs in 1%–11% of patients who have suffered blunt ocular trauma, with a mean rate of 4% [3]. It can also occur as a complication of anterior segment surgery. Any surgical procedure involving iris manipulation could cause iatrogenic cyclodialysis. In one of the largest case-series to date, 7/52 cases of cyclodialysis occurred after ocular surgery (phacoemulsification, trabeculectomy, or vitrectomy) [4]. Direct communication between the anterior chamber (AC) and the ciliochoroidal space permits excessive drainage of the aqueous humor, resulting in chronic hypotony. Its clinical manifestations include visual loss, corneal edema, shallowing of the AC, refractive changes, cataract, choroidal effusion or detachment, retinal and choroidal folding, optic disk swelling, venous tortuosity, and maculopathy [5].

When cyclodialysis is found, the cleft should be closed as quickly as possible. Several methods have been reported to close cyclodialysis, such as: medical treatment (steroids and atropine) [6]; laser treatment [7]; cryocoagulation [8]; diathermy [1]; direct cyclopexy [9]; scleral buckling [10]; and combined vitrectomy, cryotherapy, and gas tamponade [11]. In recent years, internal tamponade with a capsular tension ring (CTR) [2], implantation of an intraocular lens (IOL) into the sulcus [12, 13], or a combination of CTR and IOL implantation [14, 15] have been proposed to treat traumatic cyclodialysis, and these methods had satisfactory curative effects. Here, we present a case of cyclodialysis and chronic hypotony after trabeculectomy that was successfully treated by cataract surgery and insertion of a Cionni-modified capsular tension ring (MCTR) into the ciliary sulcus.

## Case presentations

### Clinical features of the case

**Fig. 1:**
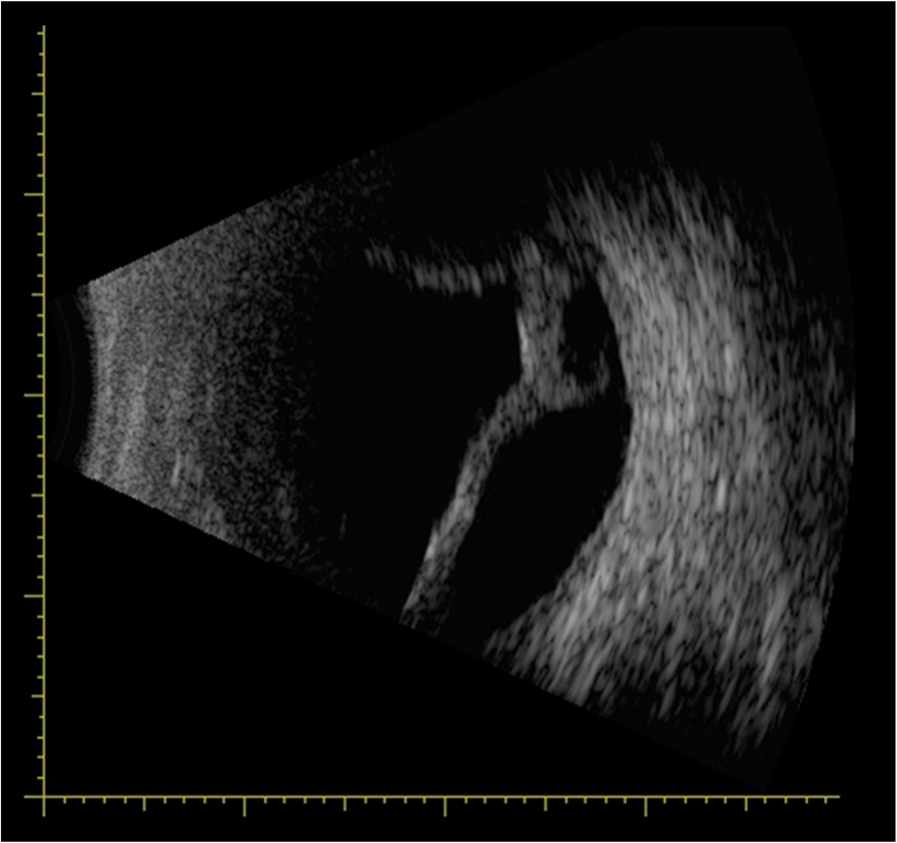
Preoperative B-scan ultrasonography showing 360 °choroidal detachment

**Fig. 2:**
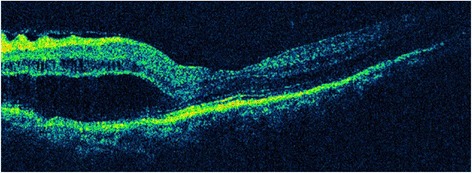
Preoperative optical coherence tomography showing choroidal detachment and the presence of retinal folds

**Fig. 3:**
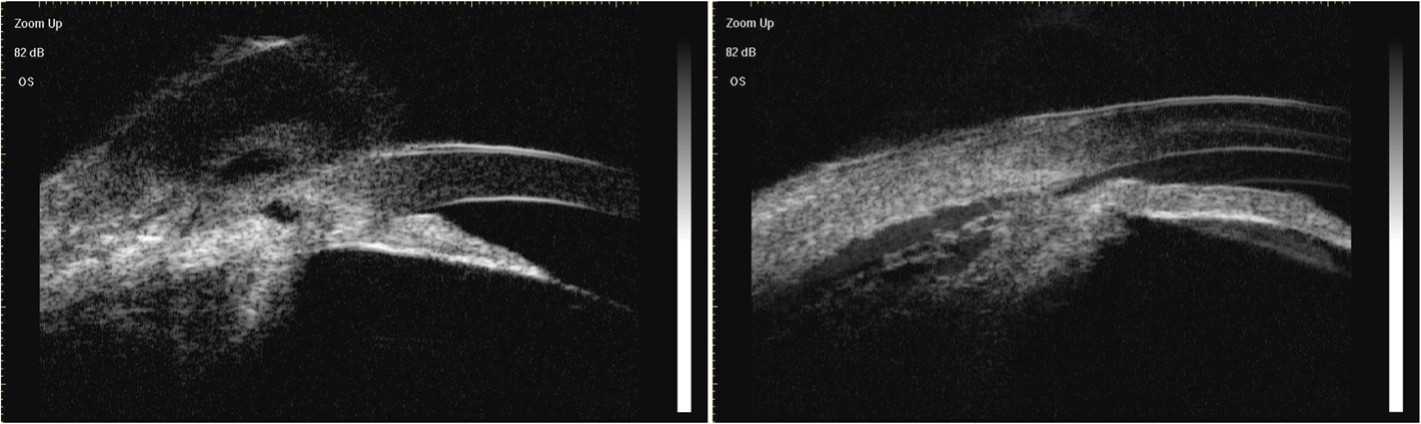
Preoperative ultrasound biomicroscopy images showing 360 ° detachment of the ciliary body and a suspected cyclodialysis in the trabeculectomy incision

A 64-year-old man complaining of blurred vision was referred to the Eye and ENT Hospital of Fudan University after trabeculectomy of the left eye 3 months earlier. His left visual acuity was 20/2000. Slit-lamp examination revealed corneal edema, a shallow AC, and posterior synechia. The lens was graded C3N3P3 according to the Lens Opacities Classification System III. The shallow AC limited gonioscopic examination. Additionally, fundus examination was not feasible owing to corneal edema and cataract. His IOP was 6 mmHg. B-scan ultrasonography revealed 360 ° choroidal detachment (Fig. 1). Optical coherence tomography (OCT5000, Carl Zeiss Meditec, Dublin, CA, USA) also revealed choroidal detachment and the presence of retinal folds (Fig. 2). Ultrasound biomicroscopy (UBM) (MD-300 L, MEDA, Tianjin, China) was performed and revealed an AC depth of 1.16 mm, 360 ° detachment of the ciliary body, suspected cyclodialysis in the trabeculectomy incision, anterior displacement of the iris–lens diaphragm, and disappearance of the posterior chamber (Fig. 3). Owing to persistent cyclodialysis and dense cataract, the patient was scheduled for surgical treatment of his left eye.

### Surgical procedure

**Fig. 4:**
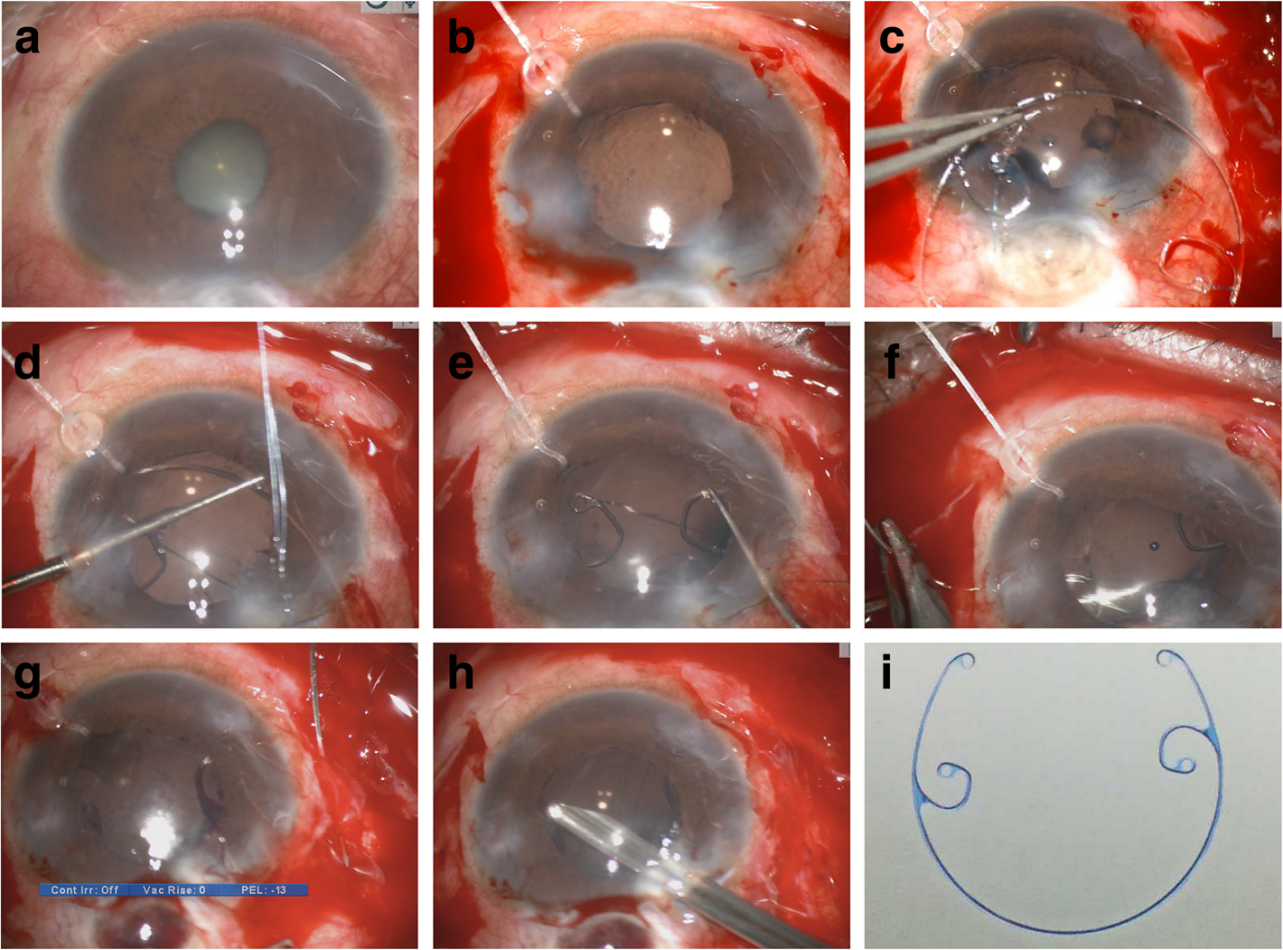
Intraoperative microscopic images. **a**: Image showing cyclodialysis combined with cataract after trabeculectomy. **b**: Filling the anterior chamber with Duovisc after routine cataract surgery to prepare for MCTR implant. **c**: A Morcher Type 2 L CTR with two eyelets preset with 10–0 polypropylene. **d**: MCTR was inserted into the ciliary sulcus. **e**: The position of the Cionni-modified capsular tension ring (MCTR) was adjusted to aim the maximum focal point of MCTR at the site of the most severe cyclodialysis. **f**:The MCTR was sutured with 10–0 polypropylene to the sclera 1 mm posterior to the corneal limbus. **g**: The suture needle was passed around the interlamellar sclera. **h**: Residual viscoelastic material was removed. **i**: A Morcher Type 2 L CTR, dimension: 11 mm (13 mm when it was stretched)


After applying retrobulbar anesthesia, a 2.6-mm superior clear corneal tunnel incision was made. A 0.9-mm-wide paracentesis was performed to insert the chopper. Subsequently, Duovisc (Alcon Laboratories Inc., Fort Worth, TX, USA) was used to maintain the AC, and continuous curvilinear capsulorhexis and hydrodissection were performed. Endocapsular phacoemulsification of the nucleus was performed using the phaco chop technique and cortical aspiration with a Centurion phacoemulsifer (Alcon Laboratories Inc.). Phacoemulsification was performed under a low flow rate with an infusion bottle height of 70 cm. The AC and capsular bag were filled with Duovisc. A foldable IOL was implanted into the capsular bag using an injector cartridge system. The viscoelastic material behind the IOL was removed using a hand-held irrigation/aspiration device. The anterior chamber was refilled with Duovisc. A 13-mm Morcher Type 2 L CTR (Morcher GmbH, Germany), an MCTR with two eyelets preset with 10–0 polypropylene (Alcon Laboratories Inc.), was then inserted into the ciliary sulcus (Fig. 4b). The position of the MCTR was adjusted to aim its maximum focal point at the site of the most severe cyclodialysis after trabeculectomy (Fig. 4c). Two conjunctival periotomies were then performed at the positions of 1 and 2 o’clock from the filtering bleb. The MCTR was sutured with 10–0 polypropylene to the sclera 1 mm posterior to the corneal limbus with one pass at 8:30 o’clock through the eyelet of the CTR and another pass through the 2 o’clock position (Fig. 4d). The suture needle was passed around the interlamellar sclera four times (Fig. 4e). The suture was tightened and cut. The end of the suture was spontaneously retracted to the interlamellar sclera. The residual viscoelastic material was removed (Fig. 4f) and the corneal wound was sutured. Balanced salt solution was injected through the paracentesis to maintain the AC. At the end of surgery, the wound was checked and found to be watertight (Additional file 1: Clip 1).

Additional file 1: Operation video. (AVI 41700 kb)


Postoperatively, the patient was treated with topical 0.1% Tobradex (Alcon Laboratories Inc.) and 0.1% pranoprofen (Sumika Finechem, Osaka, Japan) three times daily for 1 month, followed by weekly tapering.

### Results

**Fig. 5:**
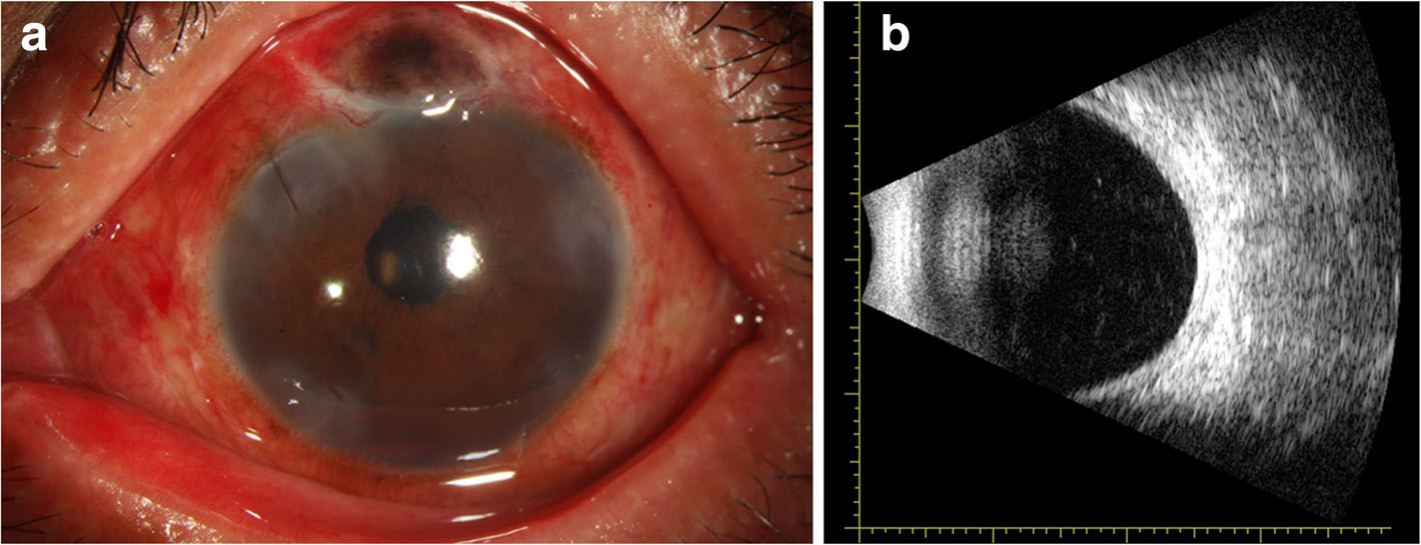
**a**: Clinical photograph of the patient showing conjunctival congestion and local corneal edema on postoperative day 1. **b**: B-scan ultrasonography showing the absence of choroidal detachment on postoperative day 1

**Fig. 6:**
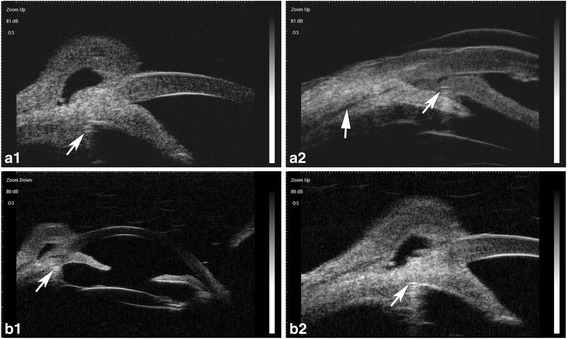
**a1** and **a2**: Ultrasound biomicroscopy images taken 2 months after surgery show that the Cionni-modified capsular tension ring is stable in the ciliary sulcus (white arrow in **a1** and right arrow in **a2**) and the cyclodialysis cleft has closed. However, 360 ° shallow ciliary body detachment is still apparent (left arrow in **a2**). **b1** and **b2**: Ultrasound biomicroscopy images taken 3 months after surgery show a deep anterior chamber, pseudophakia, location of the Cionni-modified capsular tension ring in the ciliary body (white arrow), and disappearance of the cyclodialysis

**Fig. 7:**
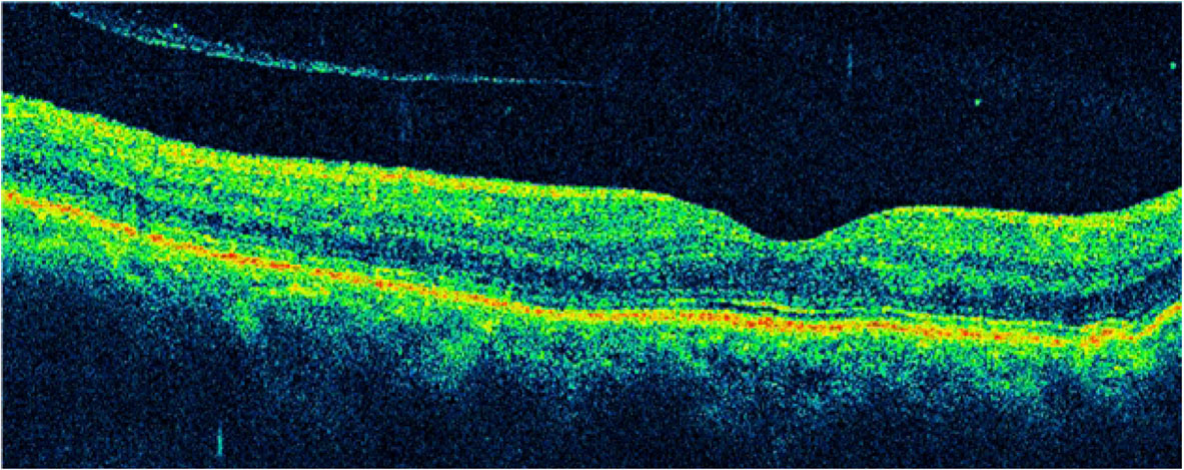
Optical coherence tomography image taken 3 months after surgery showing disappearance of choroidal detachment and a normal retinal anatomy

The patient’s visual acuity improved to 20/400 on the first day after surgery. However, his IOP was 40 mmHg, which decreased after administration of Brinzolamide and Timolol Maleate Eye Drops (Alcon Laboratories Inc.), Brimonidine Tartrate Eye Drops (Allergan Sales LLC), Bimatoprost Ophthalmic Solution (Allergan Sales LLC) twice daily. It is notable that B-scan ultrasonography revealed that the choroidal detachment had disappeared on postoperative day 1 (Fig. 5b). Two months after surgery, his visual acuity was 20/50 and the IOP was 8.2 mmHg. Slit-lamp examination revealed a clear cornea, mild conjunctival congestion, and a deeper AC. UBM 2 months after surgery showed that the AC depth was 3.36 mm, the MCTR was stable in the ciliary sulcus, the IOL was located in the center of the capsular bag, and the cyclodialysis cleft was closed. However, 360 ° shallow ciliary body detachment was evident at 2 months after surgery (Fig. 6a1, a2), but this was absent on UBM performed at 3 months after surgery (Fig. 6b1, b2). At 3 months after surgery, the best-corrected visual acuity was 30/50 with a refractive correction of −1.75DS/−1.00 DC. His IOP had recovered to 16 mmHg. Gonioscopy showed that the cyclodialysis had disappeared. Optical coherence tomography showed that the retinal anatomy was normal at 3 months after surgery (Fig. 7).

## Discussion

The clinically common complications of persistent cyclodialysis are cataract, choroidal effusion, hypotonic maculopathy, and reduced vision [13]. The vision loss is generally associated with hypotonic maculopathy [16] and serious cataract. However, some studies have reported significant recovery in visual function many years after the onset of hypotony, and the longest reported interval was 30 years [17]. It seems likely that visual acuity is mainly determined by the presence of concomitant pathologies (e.g. macular scars and retinal detachment) rather than the duration of hypotony or the extent of cyclodialysis [18]. Therefore, to address the patient’s condition, it is better to close the cyclodialysis as soon as it is found.

To our knowledge, at least 20 different techniques have been proposed for treating cyclodialysis, including medical therapy, laser therapy, diathermy, cryocoagulation, surgery, and combinations of these methods. Ophthalmologists should choose the most appropriate treatment according to the patient’s clinical condition. Ormerod initially proposed an algorithm for the management of cyclodialysis in 1991 [19]. Gonzalez-Martin-Moro et al. updated the treatment algorithm in 2016 [4]. In the first step of treatment, they recommended conservative therapy with atropine and steroids. If ocular hypotonic maculopathy persisted for 2 months after stopping steroids, then invasive or semi-invasive methods should be performed. In the absence of concomitant pathology, the treatment selection should be guided by the size of the cleft. In eyes with a small cleft (< 3 clock hours), semi-invasive laser therapy should be performed. If medical treatment is ineffective in medium-sized clefts (3–6 clock hours), or if medical and laser treatment have failed in eyes with a small cleft, then direct cyclopexia should performed. In large clefts (> 6 clock hours), posterior segment surgery seems to be the safest option. In eyes with a concomitant pathology, treatment methods that simultaneously address both pathologies should be chosen (vitrectomy in eyes with retinal pathology and cataract surgery in eyes with significant cataract) [4].

Our patient underwent trabeculectomy 3 months earlier that left a filter bleb above the corneal limbus. Direct cyclopexia was expected to do more harm than good in this case for several reasons. First, direct cyclopexia would necessitate opening the filter bleb to expose the suspected cyclodialysis cleft, destroying the filtration function of the bleb after surgery. Second, direct cyclopexia could cause severe intraoperative hypotony and other potential complications, such as intraocular hemorrhage, endophthalmitis, vitreous loss, retinal detachment, and wound dehiscence [20]. Third, suturing a hypotonic eye is challenging, requiring careful and prolonged surgery. Finally, the time to recover from this procedure might be quite long.

Although internal tamponade using an MCTR and insertion of an IOL into the ciliary sulcus to close traumatic cyclodialysis was reported 10 years ago, this is the first time that this approach has been used to treat iatrogenic cyclodialysis, specifically in a patient after trabeculectomy, and we improved this method in our case. We rotated the MCTR to ensure the maximum focal point was aligned with the site of the most severe cyclodialysis after trabeculectomy for the greatest tamponade effect. Before we fixed the sutures, the surgeon pulled the sutures carefully to ensure the MCTR tamponade was not too tight or too loose in the ciliary sulcus. The tightness of the suture was determined based on the surgeon’s experience. The MCTR was sutured to the corneal limbus with one pass through the eyelet at 8:30 o’clock and another pass through the 2:00 o’clock position, so the angle between the two sutures was <180 °. The tamponade effect of the MCTR at its maximum focal point is stronger at this angle than at an angle of 180 °.

MCTRs with two eyelets can be sutured in place more stably than MCTRs with one eyelet, as used by Yuen et al. in 2006 [2]. If the posterior chamber IOL is placed in the ciliary sulcus without suturing, as in prior reports [12–14], the tamponade effect cannot be controlled by the surgeon, and this effect could be lost or attenuated if the IOL is rotated spontaneously or by postoperative factors. In 2016, Gupta et al. [15] reported a case in which they fixed the sulcus using a Cionni ring, and a posterior chamber IOL was placed in the ciliary sulcus to close the cyclodialysis. Although this method increased the stability and tamponade effect of the MCTR, it increased the surgical difficulty and the risk of postoperative complications such as erosion, hemorrhage, and severe inflammation.

With recent improvements in surgical skills, as mentioned above, it seems that tamponade with an MCTR will be more effective in our case. In addition, we could treat the cataract and avoid secondary surgery. Compared with other methods already used to treat cyclodialysis, our method is potentially more effective, easier to perform, reduces operation time, causes less trauma, and shortens recovery time. The surgical method can be adjusted according to the patient’s specific condition. For example, in patients with zonular loss, the surgeon can insert another CTR in the capsular bag to stabilize the lens, as we have performed in an unreported case. This surgery can be used for traumatic and iatrogenic cyclodialysis, especially in patients with cyclodialysis and cataract.

Satisfactory closure of the cyclodialysis is not only dependent on the moderate internal compression elicited by the MCTR but is also on the severity of postoperative inflammation. Hypertony on postoperative day 1 is thought to be due to the recovery of aqueous humor production by the ciliary body and partial functional recovery of the trabecular meshwork [14]. The IOP returns to normal after full functional recovery of the trabecular meshwork, permitting discontinuation of anti-glaucoma eye-drops. The recovery of the choroidal detachment on postoperative day 1 was due to hypertension and closure of the cyclodialysis cleft. Detachment of the ciliary body had completely recovered by 3 months after surgery in our case, and we think that the suprachoroidal effusion had a high protein concentration, and took some time to be completely absorbed.

## Conclusions

In conclusion, phacoemulsification with IOL implantation and insertion of an MCTR in the ciliary sulcus is a relatively safe, effective, minimally invasive method to repair cyclodialysis in cataract patients. More cases and long-term follow-up data are needed to validate the effectiveness of this method.

## Abbreviations

AC: anterior chamber
BCVA: best corrected visual acuity
CTR: capsular tension ring
IOL: intraocular lens
IOP: intraocular pressure
MCTR: Cionni modified capsular tension ring
OCT: optical coherence tomography
UBM: Ultrasound biomicroscopy
